# Vessel co-option: a unique vascular-immune niche in liver cancer

**DOI:** 10.3389/fonc.2024.1386772

**Published:** 2024-04-26

**Authors:** Dan Yang, Shumin Dang, Zhiyi Wang, Meng Xie, Xiuling Li, Xiangming Ding

**Affiliations:** Department of Gastroenterology, People’s Hospital of Zhengzhou University, Henan Provincial People’s Hospital, Zhengzhou, Henan, China

**Keywords:** vessel co-option, tumor microenvironment, anti-angiogenic therapy, immunotherapy, drug resistance, liver cancer

## Abstract

Tumor vasculature is pivotal in regulating tumor perfusion, immune cell infiltration, metastasis, and invasion. The vascular status of the tumor is intricately linked to its immune landscape and response to immunotherapy. Vessel co-option means that tumor tissue adeptly exploits pre-existing blood vessels in the para-carcinoma region to foster its growth rather than inducing angiogenesis. It emerges as a significant mechanism contributing to anti-angiogenic therapy resistance. Different from angiogenic tumors, vessel co-option presents a distinctive vascular-immune niche characterized by varying states and distribution of immune cells, including T-cells, tumor-associated macrophages, neutrophils, and hepatic stellate cells. This unique composition contributes to an immunosuppressive tumor microenvironment that is crucial in modulating the response to cancer immunotherapy. In this review, we systematically reviewed the evidence and molecular mechanisms of vessel co-option in liver cancer, while also exploring its implications for anti-angiogenic drug resistance and the immune microenvironment, to provide new ideas and clues for screening patients with liver cancer who are effective in immunotherapy.

## 1 Introduction

Previous studies have concluded that solid tumors must form neovascularization to ensure their nutritional and metabolic requirements (1). Based on this theory, anti-angiogenic therapy (AAT) has rapidly emerged as a research focal point in tumor treatment. AAT mainly inhibits the binding of vascular endothelial growth factor (VEGF) to vascular endothelial growth factor receptor (VEGFR). On the one hand, it inhibits tumor neovascularization and promotes the normalization of vascular morphology, size, and permeability to reduce intertissued hydraulic pressure and increase oxygen (2). Vascular normalization can directly cause T-cells to flow into solid tumors and indirectly change immunosuppression by reducing, for example, alternately activated macrophages, myeloid suppressor cells, and/or regulatory T cells, thereby improving the prognosis (3). Although liver cancer is a highly vascularized tumor, clinical and preclinical data show that AAT does not significantly improve the overall survival (OS) of patients (4, 5). This suggests that the vascular system of liver cancer is much more complex than expected.

In the 1990s, Pezzella et al. first observed the phenomenon of vessel co-option (VC) in lung cancer. They showed that some lung cancers could use pre-existing vessels to promote their own growth, rather than induce new angiogenesis (6, 7). Since then, researchers have successively found this phenomenon in various primary and metastatic tumors, such as liver cancer, glioblastoma, kidney cancer, and pancreatic cancer (8–10). This phenomenon challenges the hypothesis that tumor growth must require new blood vessel formation. Distinguishing from angiogenic tumors, VC promotes tumor growth by “hijacking” the blood vessels already present in the paracancerous tissue (11). Further studies have shown that VC enhanced tumor cell motility and surrounding tissue infiltration, leading to poorer prognosis in some advanced-stage cancer patients (8). In addition, VC is independent of endothelial cell proliferation, and kinds of literature have shown that VC is related to AAT resistance in liver cancer (12, 13).

The tumor immune microenvironment is highly heterogeneous and can be regulated by tumor vascularization mode. Different from angiogenic tumors, VC shows a unique vascular-immune niche. The different states, functions, and distribution of many kinds of immune cells, such as T-cells, tumor-associated macrophages (TAMs), hepatic stellate cells (HSCs), and neutrophils constitute the inhibition state of the VC immune microenvironment, which affects the efficacy of immunotherapy for liver cancer.

In this review, we conducted a comprehensive analysis of the available evidence, clinical prognosis, and underlying molecular mechanisms associated with VC in liver cancer. Additionally, we investigated its implications in terms of resistance to anti-angiogenic drugs and its impact on the immune microenvironment status. Furthermore, we engaged in a comprehensive exploration of therapeutic approaches for VC, aiming to identify appropriate treatment regimens and offer novel therapeutic insights for individuals afflicted with liver cancer.

## 2 Vessel co-option in liver cancer and clinical prognosis

### 2.1 Primary liver cancer

**Figure 1:**
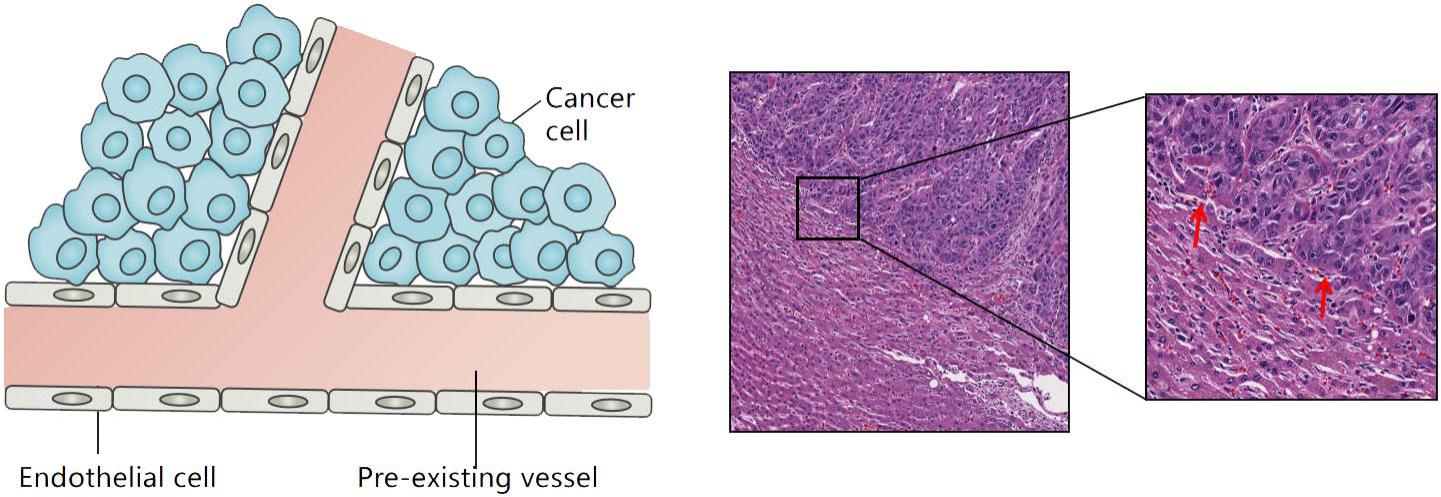
Tumor growth patterns associated with vessel co-option in liver cancer: cancer cells grow within the liver cell plates while replacing the pre-existing hepatocytes. The red arrow refers to the co-option vessels.

Early small HCC (diameter less than 2 cm) exhibits two distinct vascularization patterns. In the early stage of nodular HCC, angiogenesis is often induced to meet its own occurrence and development. However, diffuse HCC demonstrates a unique “replacement” growth pattern where cancer cells do not disrupt normal liver tissue but rather “hijack” hepatic sinusoidal vessels or portal tracts to achieve vascular colonization by replacing normal hepatocytes in the hepatic plate (14) (**Figure 1**).

VC can also be observed in advanced HCC. With the continuous invasion of cancer cells, hepatic sinusoidal vessels or portal venous bundles are gradually integrated into the tumor tissue (11). In addition, a relatively rare VC pattern known as “sinus” VC mode exists in rapidly progressive end-stage HCC. In this mode, cancer cells are confined to the luminal surface of hepatic sinusoid vessels. Autopsy studies of early liver cancer suggest that VC can be observed in 60% of liver cancers (15). Kuczynski et al. showed that the proportion of VC in sorafenib-resistant liver cancer tissues was as high as 75% (16).

### 2.2 Secondary liver cancer

Liver metastases can also utilize VC to achieve blood supply. The vascularization pattern of liver metastasis is influenced by the primary tumor. Two independent studies have confirmed that VC can be observed in more than 90% of breast cancer liver metastases (10, 17). In patients with colorectal cancer liver metastasis (CRCLM), 47% of them use VC, while the remaining patients still exhibit angiogenic characteristics (18). A study has confirmed that CRCLM patients with VC have a high positive rate of resection margin, high recurrence rate, and poor prognosis after hepatectomy (18). In addition, a series of clinical studies have shown that VC exists widely in patients with liver metastasis after AAT, and these patients tend to exhibit poor response to angiogenesis inhibitors (10, 19, 20).

## 3 Molecular mechanisms of vessel co-option in liver cancer

The determinants influencing the selection of vascularization mode in liver cancer and the specific mechanism underlying VC remain elusive. What is worth paying attention to is what role VEGF plays in VC. Studies have shown that VEGF interacts with angiopoietin-2 (Ang-2)/Tie-2 and dynamically participates in the VC process (21). When the level of VEGF is low, the vascular endothelial cells involved in co-option highly express Ang-2. Ang-2 interrupts the interaction between endothelial cells and surrounding Sertoli cells and endothelial cell apoptosis by binding to Tie-2, resulting in obvious vascular degeneration (21–23). With the increase of VEGF-induced expression, Ang2/Tie2 induced endothelial cell proliferation and triggered neovascularization. VEGFR-2 is the main receptor of VEGF. When it is inhibited, the tumor changes from angiogenesis to VC (16, 24). Therefore, VC can be regulated by VEGF/VEGFR-2 and Ang-2/Tie-2 pathways. In addition, existing studies have shown that VC encompasses diverse mechanisms encompassing cancer cell motility and adhesion, epithelial-mesenchymal transition (EMT), and metabolic reprogramming. Therefore, we will discuss the mechanism of VC in liver cancer from these aspects.

### 3.1 Cancer cells motility and adhesion

**Figure 2:**
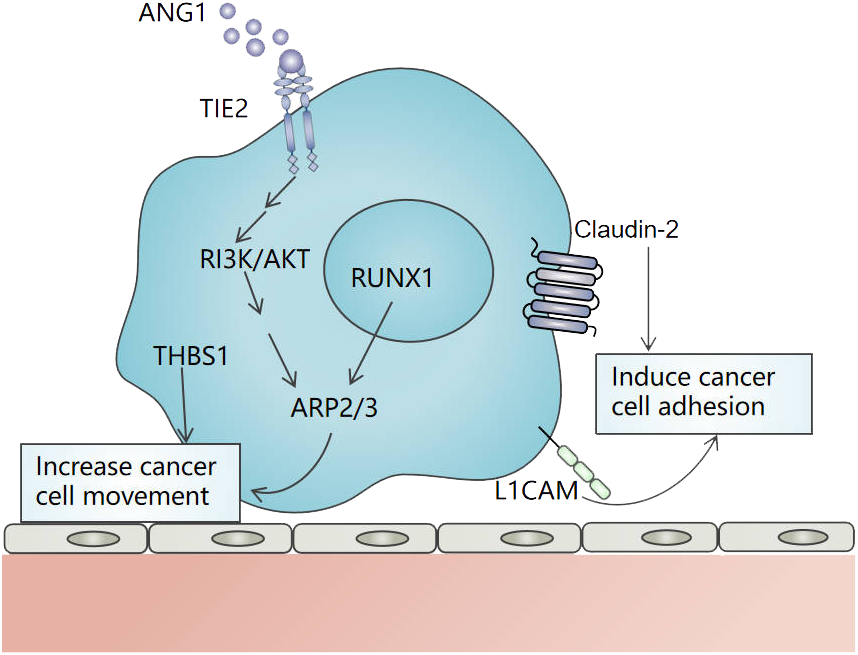
The mechanism of movement and adhesion of cancer cells in vessel co-option in liver cancer. ARP2/3 and THBS1 induce cancer cells movement, and ARP2/3 is regulated by RUNX1 and Ang-1/Tie-2/PI3K/AKT pathway. L1CAM and Claudin-2 promote the adhesion of cancer cells to the vascular basement membrane or the endothelial cells of pre-existing blood vessels. .

In theory, VC depends on the cancer cells motility and adhesion. Motility refers to cancer cells invading adjacent tissues, approaching or wrapping co-option vessels. Adhesion refers to cancer cells adhering to vascular basement membrane or endothelial cells after arriving at blood vessels. Inducing cancer cell movement is the first stage in the establishment of VC, which enables cancer cells to invade their neighboring tissues and co-opt the pre-existing vessels. In VC of liver cancer, actin-related protein 2/3 (ARP2/3) and thrombospondin 1 (THBS1) are important in inducing cancer cells motility. ARP2/3 can mediate the actin nucleation and promote the movement of cancer cells toward adjacent tissues (25, 26). Frentzas et al. demonstrated that knockdown ARP2/3 can suppress VC in a preclinical model of advanced liver metastasis (10). In CRCLM, overexpressed angiopoietin-1 (Ang-1) can bind to Tie-2 and promote VC through the PI3K/AKT pathway (27). In addition, Runt-associated transcription factor-1 (RUNX1) is overexpressed in cancer cells that replace lesions. It drives cancer cells movement through ARP2/3 to achieve VC (28). A study has shown that the expression of THBS1 in VC is higher than that in angiogenic tumors (29). In CRCLM, THBS1 induces cancer cells movement and promotes VC (29) (**Figure 2**). In addition, CDC42, CD44, EGFRvIII, CXCR4-CXCL12, and Olig2-Wnt7a have been found to promote the movement of cancer cells in other organ tumors (30–33).

In VC, cancer cells often need to adhere to the vascular basement membrane or the endothelial cells of pre-existing blood vessels through a variety of adhesion molecules. Claudin-2, L1 cell adhesion molecule (L1CAM), and so on are involved in VC. Claudin is an important component involved in tight junctions between cells. Tabaris et al. have shown that Claudin-2 participates in the adhesion of colon cancer cells to hepatocytes and is highly expressed in VC. Its high expression is related to poor OS and progression-free survival (PFS) in CRCLM patients (34). L1CAM is a highly expressed cell surface glycoprotein in metastatic tumors (35). In uveal melanoma liver metastasis, L1CAM enables cancer cells to attach and utilize existing blood vessels in the liver (36). The latest research shows that the overexpression of αVβ3-integrin is also an important part of VC. In CERCLM, the overexpression of Alanine-Serine-Cysteine 2 (ASCT2) can promote the expression of αVβ3-integrin in tumor cells, and then activate the αVβ3/FAK/PI3K/AKT signal pathway, thus promoting VC (24). Integrin families such as β1-integrin, α3-integrin, β4-integrin, and β6-integrin also have been found to promote the adhesion of cancer cells in other organ tumors during VC (37–39). In addition, α3β1- integrin may be involved in the formation of VC in liver cancer. It not only promotes the migration and invasiveness of HCC but also has anti-angiogenic properties when interacting with a 12-residue peptide of thrombospondin 1 (40–42). The action mechanism of α3β1-integrin in VC needs to be further studied.

### 3.2 EMT

EMT means that epithelial cells are transformed into cells with stromal phenotype. It shows changes in cell morphology, polarity, and phenotype, and decreased intercellular adhesion, accompanied by abnormal cell signal pathways and gene expression, increasing tumor invasion and metastasis (43). EMT is characterized by the deficiency or decreased expression of the epithelial marker E-cadherin, overexpression of E-cadherin repressors such as Zeb-1, Zeb-2, Twist, Snail, and Slug, and increased expression of mesenchymal markers such as vimentin and N -cadherin (44–46). When cancer cells approach pre-existing blood vessels, their growth depends on invading healthy tissue from neighboring blood vessels. Therefore, cells close to VC lesions must undergo phenotypic changes, which may be displaced by cancer cells. In a VC-dependent CRCLM, Rada et al. found that the expression of E-cadherin decreased and vimentin increased significantly in hepatocytes in close contact with cancer cells (47). Similarly, in another study, the expression of EMT markers including vimentin, ZEB1, and ZEB2 increased significantly in the sorafenib-resistant group, resulting in the infiltration of cancer cells into the surrounding liver tissue and the formation of VC (16). In summary, these dates strongly suggest the correlation between the EMT process and VC. At present, the potential molecular mechanism of the role of EMT in VC is not clear, and further research is needed.

### 3.3 Metabolism reprogramming

Metabolism reprogramming is one of the characteristics of malignant tumors, including glycolysis, oxidative phosphorylation, amino acid metabolism, fatty acid metabolism, and nucleotide metabolism. It provides material and energy bases for tumor growth, proliferation, invasion, and metastasis. Studies suggest that AAT usually induces hypoxia microenvironment before tumor-acquired drug resistance, which is beneficial to the transformation of tumor metabolism to glycolysis and glutamine metabolism (48–50). In VC, the tumor tissue showed enhanced glycolysis and pentose phosphate pathway activation (51). The pentose phosphate pathway has the function of anti-oxidation and defense and has a higher resistance to reactive oxygen species. It can lead to the survival benefit of cancer cells and is related to the malignant progression and poor prognosis of tumors (52). Further study showed that glutamine transporter ASCT2 was overexpressed in bevacizumab or regorafenib-resistant HCT116 CRCLM xenograft. ASCT2 promoted VC by inducing tumor EMT, and cell proliferation, and promoting the recruitment of Gr-1+ myeloid-derived suppressor cells (MDSCs) and F4/80 ^+^ TAMs (24). In addition, in non-small cell lung cancer VC, the expression of genes involved in mitochondrial regulation was up-regulated, and the levels of oxidative phosphorylation and mitochondrial biogenetic transcripts such as GPI, NDUFB6, ANXA7, and PRSS15 are higher. This suggests that, in lung cancer VC, the metabolic transition from glycolysis to oxidative phosphorylation may occur (53). These studies suggest that metabolism reprogramming may be involved in various organs of VC, but the specific mechanism in VC formation is not clear.

## 4 Vessel co-option meditates AAT resistance in liver cancer

The resistance of VC to AAT can be either intrinsic (observed from the beginning of treatment) or acquired (observed after AAT treatment). In theory, since VC is independent of endothelial cell angiogenesis, VC in liver cancer may show resistance to AAT from the beginning of treatment. In a clinical study of bevacizumab combined with chemotherapy in the treatment of CRCLM, the researchers observed that patients with angiogenic CRCLM had a better response to treatment. Patients with a poor response or no response mainly showed VC growth pattern (10). Another independent study also confirmed that bevacizumab could significantly inhibit angiogenesis but did not affect the co-option vessels in HCC (19). In addition, another mechanism of VC-mediated intrinsic drug resistance in AAT is stable fluid shear stress. Fluid shear stress is the tangential friction force exerted by the stratosphere on the surface of vascular endothelium, which is affected by blood viscosity, blood flow, vascular diameter, and so on (54). When fluid shear stress decreases, it will lead to abnormal growth of blood vessels, an increase in tumor activity, invasion and migration, etc (55–57). In angiogenic tumors, neovascularization is usually immature, vascular walls are fragile, endothelial cells and pericytes are arranged irregularly, resulting in incoherent blood leakage and perfusion. High permeability tumor vessels promote the entry of plasma and proteins into the surrounding stroma and increase blood viscosity in the tumor microenvironment (58). The blood flow is irregular and chaotic, and the blood flow velocity and blood flow are unevenly distributed. There are more vascular branches in the process of forming the reticular structure of tumor vessels, so the flow velocity of blood in tumor vessels is lower than that in normal blood vessels. However, in VC tumors, the co-option vessels have dense pericyte coverage and vascular endothelial cell connections, ensuring normal blood circulation and no leakage (21, 59). Therefore, the shear stress of angiogenic tumors is lower than that of vessel co-opting tumors, which leads to an increase in tumor activity, proliferation, invasion, and metastasis. Anti-angiogenic drugs restore the functional and morphological characteristics of tumor vessels to a normal state, reduce leakage, reduce curved blood vessels, make basement membrane more normal, and increase pericyte coverage, resulting in increased shear stress and promote tumor cell apoptosis (60). Because angiogenic tumors use pre-existing mature vessels to grow, the shear stress does not change significantly, so the effect of “vascular normalization” of anti-angiogenic drugs is general.

The resistance of VC to AAT may also be attributed to its adaptive ability acquired through the inhibition of tumor angiogenesis. It is speculated by researchers that this acquired drug resistance might be associated with the transition in the tumor’s vascularization pattern from angiogenesis to VC (8). Kuczynski et al. found that in sorafenib-resistant liver cancer tissues, the proportion of VC was as high as 75%, while in the untreated control group, it accounted for only 23.3% (16). This finding further suggests that the vascularization mode of HCC has changed significantly from angiogenesis to VC during the process of drug resistance. These findings suggest that VC, as an important mechanism of drug resistance in AAT, poses a new challenge to clinical tumor therapy. The treatment strategy for VC needs to be further explored.

## 5 The tumor immune microenvironment in vessel co-option

### 5.1 T-cells

According to the distribution and activity of T-cells within the tumor microenvironment, the tumor can be classified into three distinct immunophenotypes: immune desert, immune-inflamed, and immune-excluded phenotypes (61). VC showed an immune desertification phenotype, and there was little T-cells infiltration in the tumor and stroma. Brunner et al. studied the immune cell density at the tumor-liver interface in 201 patients with CRCLM. They observed low levels of CD4, CD45RO, and CD8 positive T cells at the infiltrating edge of tumors using VC, as opposed to angiogenic tumors (62). Similarly, Vermeulen et al. observed that a low density of CD8-positive immune cells was present at the interface between the carcinoma tissue and the adjacent liver in VC (63). A recent study by Scherer SJ et al. has also observed this phenomenon (53). In addition, CRCLM patients using VC showed high MHC-I expression and low CD3^+^ T cell count, which was associated with the risk of early recurrence (64). To sum up, these studies suggest that VC has an immune desertification phenotype and that its level of T cell infiltration is associated with a poor response to current treatment regimens.

### 5.2 Tumor-associated macrophages

During VC, macrophages assist in cancer cell invasion. In the model of renal cancer lung metastasis model of VC, matrix-remodeling macrophages are enriched at the front of invasion. They are characterized by high expression of genes involved in extracellular matrix (ECM) remodeling (Ctsd, Ctss, Ctsb, Gpnmb, etc.), and genes supporting cancer cell invasion and migration (Spp1, Cd63, Pdpn, and Anxa1, etc.), which participate in leukocyte-endothelial cell interaction (65). For cancer cells to move to pre-existing blood vessels, they must pass through the dense stroma. Matrix-remodeling macrophages can pave the way through matrix recombination, including the degradation of existing matrix and the deposition of new matrix, assisting cancer cells to participate in VC (66). In addition, the authors also observed the enrichment of antigen-presenting cells/inflammatory macrophages on the surrounding edge of tumor nodules using VC (65). In a single-cell analysis of mononuclear phagocytes infiltrating human CRCLM, TAMs with high expression of glycoprotein nonmetastatic melanoma protein B (GPNMB) were found to be enriched at the invasive edge. The high density of GPNMB^+^ cells was associated with shorter disease-free survival and OS. Subsequent investigations revealed that GPNMB-high TAMs exerted immunosuppressive effects by strongly binding to CD8^+^ T cells through key immunosuppressive cytokines, namely IL20 and IL10 (67). In addition, Xiong et al. found that GPNMB-high macrophages ineffectively retain T cells from activating by dendritic cells due to continuous co-stimulation signals but instead exert non-effective retention. Furthermore, GPNMB-high macrophages can interact with T-cells through chemokines such as Ccl2-Cxcr3, Cxcl16-Cxcr6, and Ccl3-Ccr1 (68). These findings suggest that the overexpression of GPNMB in macrophage subsets may present a novel strategy for inhibiting VC and promoting T cell-based immunotherapy. It is time to address the knowledge gap regarding the role of TAMs in the initiation and progression of VC in liver cancer.

### 5.3 Hepatic stellate cells

HSCs are located in the disuse space and close to hepatic sinusoidal endothelial cells. They are irregular in shape and often extend several stellate processes around the hepatic sinusoids. HSCs can secrete chemokines, cytokines, growth factors, or proteases, which promote tumor growth, metastasis, angiogenesis, and immune escape, and are considered liver-specific pericytes (69–72). Ming Qi et al. found that in the CRCLM models of bevacizumab resistance, the HSCs around the co-option vessels had a high expression of fibroblast activation protein α (FAPα). FAPα induced CXCL5 secretion in HSCs and then activated CXCR2, which promoted tumor cell EMT and the recruitment of MDSCs, inhibiting the infiltration of CD8^+^ T cells (73). However, a recent single-cell RNA-seq analysis of a murine AAT-resistant lung tumor model revealed an increase in quiescent co-opted pericytes but a decrease in angiogenic/activated pericytes within the VC (65). These findings suggest that pericytes exhibit distinct states within the VC of metastasized tumors, potentially influenced by the fibrotic response of HSCs to liver injury or inflammation. Although the exact state and underlying mechanism of pericytes in VC remain inconclusive, several studies have indicated their potential role in promoting endothelial cell survival (74). This could be a significant factor contributing to the resistance observed in co-option vessels with intact structure and high pericyte coverage. Furthermore, these observations offer novel insights and avenues for targeting VC within the tumor immune microenvironment.

### 5.4 Neutrophils

Evidence of pro-tumorigenic and pro- metastatic of neutrophils has been widely studied (75–77). Palmieri et al. have observed that, compared to angiogenic lesions, a significant increase in the number of neutrophils at the tumor-liver interface and peri-tumor stroma in CRCLM lesions using VC (78). These neutrophils express high levels of lysyl oxidase-like 4 (LOXL4) (78). Further study showed that in VC, RUNX1 is highly expressed in the cancer cells, which induces the expression of transforming growth factor β1 (TGF-β1)and Ang-1 in the neighboring liver parenchyma. The overexpression of Ang1 in the hepatocytes incites the migration of neutrophils into the tumor microenvironment. However, the role of neutrophils in the development and maintenance of VC needs further research.

### 5.5 ECM

ECM is an important part of the tumor microenvironment. ECM provides the support and structure needed for cell growth and migration and contains many growth factors and cell adhesion molecules. It can regulate the growth, proliferation, migration, and metastasis of tumor cells (79). Important components of ECM, such as collagen, laminin, and matrix metalloproteinase (MMP) can interact with adhesion molecules to promote the movement of tumor cells towards existing blood vessels (80, 81). Palmieri et al. showed that the levels of MMP-2, and MMP-14 in VC-type CRCLM were significantly higher than those in angiogenic CRCLM, and the specific collagen proteins including COL10A1, COL13A1, COL14A1, and COL17A1 were significantly up-regulated (78). Activated MMP14 and MMP2 split laminin 5γ2 into motion-promoting segments 5γ2’and 5γ2x, which are an essential part of non-angiogenic tumors (59). In addition, compared with angiogenic tumors, the invasive front of uveal melanoma using VC is rich in “L1CAM and laminin vascular network” (36). Laminin is located in the basement membrane between sinus vascular channels and angiophilic melanoma cells. It binds to the highly expressed L1CAM of melanoma cells and makes cancer cells spread along the vascular channels. These studies suggest that ECM components such as laminin, collagen, and MMP may promote VC. On the other hand, VC may also affect the composition of ECM. Vessel co-opting tumors oppress existing blood vessels, resulting in deformation and hypoxia (82). Hypoxia is beneficial to the formation of type I collagen and ECM remodeling through collagen degradation by matrix metalloproteinases, thus increasing the invasiveness of cancer cells (83). In summary, fully understanding the changes of ECM in VC is beneficial to targeted therapy.

**Figure 3:**
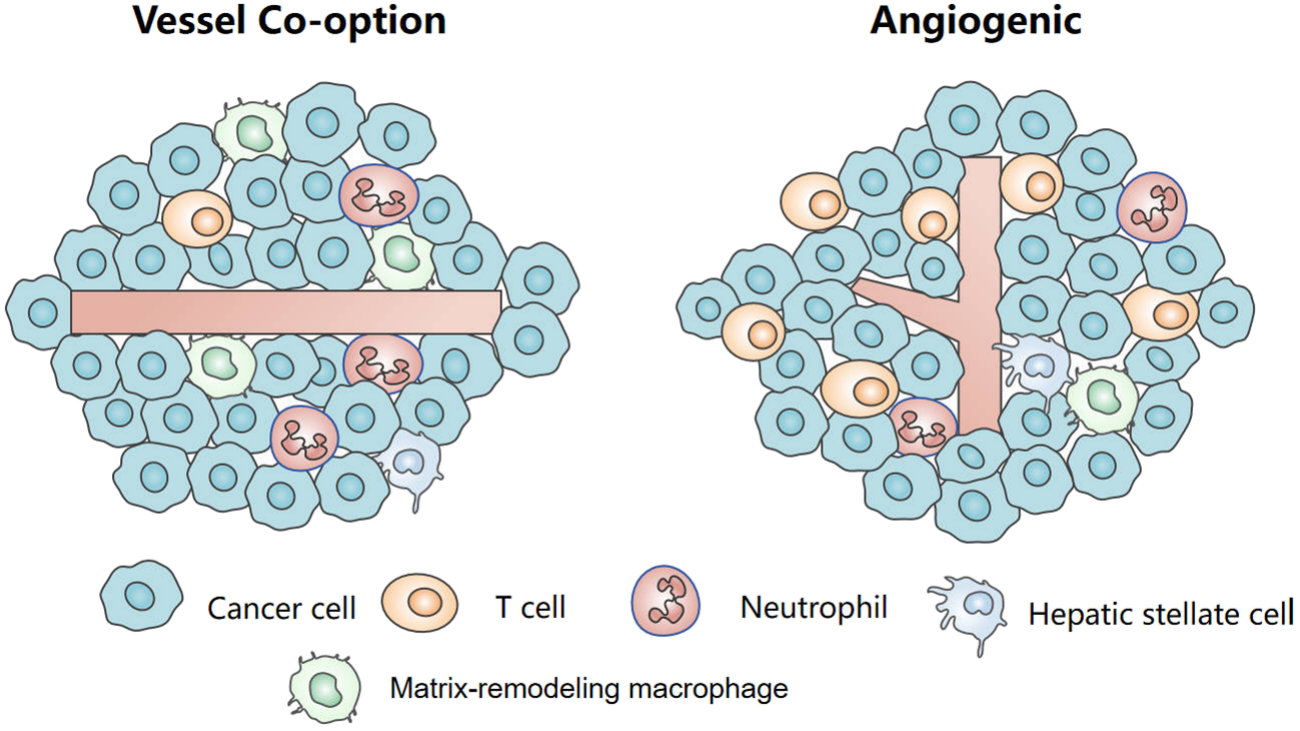
The tumor immune microenvironment in vessel co-option and angiogenesis.

In summary, VC shows a unique immune niche (**Figure 3**). A comprehensive and in-depth analysis of the composition, characteristics, and dynamic changes within the immune microenvironment in VC will facilitate the development of personalized therapy strategies for liver cancer in the future. However, currently, our understanding of the role played by the immune microenvironment in VC remains limited and necessitates further exploration.

## 6 Therapy

### 6.1 Immunotherapy

In recent years, immunotherapy represented by immune checkpoint inhibitors has shown reliable clinical efficacy in liver cancer treatment. Immune checkpoint inhibitors can alleviate the interaction between immune checkpoint proteins and their ligands, promote T cell activation and proliferation, and avoid tumor immune escape (84). However, tumors with limited levels of T-cell infiltration showed inherent resistance to immune checkpoint inhibitors (85). Studies have shown that there are only low levels of immune and/or inflammatory cell infiltration in VC liver metastases, showing the characteristics of immune “desertification” (63). Therefore, immune checkpoint inhibitors alone may not be effective in suppressing VC. Previous studies have suggested that AAT can increase tumor immune cell infiltration and enhance the efficacy of immunotherapy. Therefore, immune checkpoint inhibitors combined with AAT may enhance the efficacy of immunosuppressants in the treatment of VC within liver cancer (86).

In the study of the AAT-resistant lung tumor model in mice, M1 macrophages were highly enriched in VC (65). The enrichment of these pro-inflammatory macrophages shows that VC can activate cytotoxic T lymphocytes and lead to anti-tumor immunity. However anti-tumor immunity is not enough to reduce tumor load (65). Therefore, promoting the polarization of M1-like macrophages may be helpful to improve the prognosis of VC. In a mathematical modeling study about VC, it was found that blocking VEGF and VC at the same time could enhance tumor oxygenation and increase the abundance of M1 macrophages, thus improving the prognosis of VC (82). It provides a new idea for treating VC liver cancer.

HSCs are considered to be liver-specific pericytes and play an important role in VC in liver cancer. Ming Qi et al. confirmed that blocking the FGFBP1/FGF2/FGFR1 signal pathway can inhibit the expression of FAPα in HSCs and attenuate VC. Z-GP-DAVLBH, a FAPα-activated prodrug, selectively induces apoptosis of FAPα^+^ HSCs and destroys co-opted sinusoid vessels to overcome VC-mediated bevacizumab resistance (73). The specific role of pericytes in different organ tumors using VC is not clear, and more experiments are needed to explore.

Another concern revolves around the pivotal role of liver sinusoidal endothelial cells (LSECs) in tumor immunity as a crucial component of VC. LSECs can express PD-L1. PD-L1 interacts with PD-1 on T cells, which hinders T cell function and suppresses its anti-tumor activity, which mediates liver immune tolerance (87–90). Moreover, LSECs also employ other inhibitory or immunomodulatory molecules such as Fas ligand, LSECtin, and IL-10 to effectively regulate T-cells function (91). Furthermore, both *in vivo* and *in vitro* studies demonstrate that LSECs can facilitate the conversion of Treg into functional suppressor cells (92). In summary, LSECs possess robust immunomodulatory capabilities and play a pivotal role in maintaining immune tolerance. Ongoing evaluations are currently underway for antibodies targeting PD-L1 expression in LSECs, such as durvalumab (93). Consequently, future research endeavors should focus on elucidating the expression of PD-L1 within LSECs and its significance within the vascular-immune niche in VC.

### 6.2 Targeted therapy

Targeting VC especially inhibits tumor cell movement and adhesion, is the current research hotspot. Preclinical studies of CRCLM have shown that knockout of ARP2/3 can inhibit the movement of tumor cells, thus suppressing VC (10). In addition, Tabaris et al. have shown that the ability of colon cancer cells lacking Claudin-2 to metastasize to the liver decreases, and VC production decreases (34). It has been reported that L1CAM can promote the expression of adhesion molecule β1 integrin and up-regulate ARP2/3 to promote cell motility. At the same time, L1CAM can also inhibit the maturation of the tumor vascular system. Therefore, in theory, inhibition of L1CAM can not only suppress VC by preventing cancer cells motility and adhesion but also increase the efficacy of chemotherapy or immunotherapy inducing vascular normalization (82, 94). However, this conjecture still needs to be proved by further experiments.

In the process of EMT, cancer cells lose their epithelial characteristics and acquire mesenchymal characteristics. As a result, the adhesion between tumor cells decreased and the migration increased, which is beneficial to the formation of VC. Research shows that cancer cells induce EMT through overexpression of TGF-β, which in turn promotes VC (47). At the same time, it has been reported that TGF-β1 can stimulate hepatocytes to produce EMT in a dose and time-dependent manner (95). Therefore, inhibition EMT driven by TGF-β1 is one of the strategies for the treatment of AAT-resistant tumors.

Glutamine is the most abundant amino acid in the blood and plays an important role in providing carbon and nitrogen for synthetic metabolism. In CRCLM, the combination of glutamine transporter ASCT2 inhibitor and bevacizumab or regorafenib attenuated VC. Compared with bevacizumab or regorafenib alone, they significantly prolonged the OS of mice (24). In short, the therapeutic strategy of inhibiting glutamine transporter has opened up a new approach to overcome VC-mediated AAT drug resistance.

### 6.3 Chemotherapy

Some scholars have proposed that VC exposes cancer cells to a unique niche and induces chemotherapy resistance. Lu et al. observed that in vessel co-opting tumors, endothelial cells produce ligand Jagged-1, which induces stem cell phenotype in cancer cells, which may affect the efficacy of chemotherapy (96). In addition, under the background of the unique dual blood supply of the liver, cancer cells using co-opted vessels can survive from hepatic artery chemoembolization because of co-opted with the portal vein system instead of the hepatic artery (97). On the contrary, some scholars believe that VC has a relatively normalized vascularization system, which makes tumors more likely to benefit from chemotherapy. In addition, chemotherapy combined with AAT may inhibit VC. For example, in the VC-dependent metastatic triple-negative breast cancer model, the combination of topotecan and pazopanib significantly enhanced the antitumor activity and prolonged the OS of patients compared with topotecan alone (98).

### 6.4 Other treatments

Tumor hypoxia microenvironment will affect the effect of radiotherapy, so the application of AAT to promote vascular normalization to improve the effect of radiotherapy has been a long-awaited clinical strategy. According to this idea, the combination strategy based on radiotherapy is more expected in VC. In addition, some scholars have begun to study the effect of metformin on VC of liver cancer in recent years. A study showed that CRCLM patients treated with metformin have fewer co-option vessels than unused patients (99). At the same time, Li et al. have shown that metformin can improve the chemotherapy resistance of non-angiogenic colorectal cancer by increasing microvessel density and restoring vascular function and maturity (100). This suggests that metformin may play a role in the treatment of VC, but the specific mechanism remains to be further studied.

## 7 Summary

VC is an emerging and captivating domain in the realms of angiogenesis and tumor biology. The emergence of VC-mediated AAT resistance and immunosuppression in liver cancer presents a novel challenge for clinical interventions. Nevertheless, our current comprehension of VC in liver cancer remains nascent. Understanding the theoretical underpinnings behind VC-induced AAT resistance, and delving into the intricate landscape of the tumor immune microenvironment within VC may yield fresh insights and innovative approaches toward precision-based therapies for liver cancer.

## Author contributions

DY: Writing – original draft, Writing – review & editing, Investigation, Software. SD: Writing – review & editing. ZW: Writing – original draft. MX: Writing – original draft, Writing – review & editing. XL: Writing – review & editing. XD: Funding acquisition, Resources, Supervision, Writing – original draft, Writing – review & editing.
